# 3D whole heart imaging in severe funnel chest and non-compaction cardiomyopathy

**DOI:** 10.1007/s10554-020-02030-0

**Published:** 2020-09-22

**Authors:** M. Polacin, P. Biaggi, H. Alkadhi, S. Kozerke, R. Manka

**Affiliations:** 1 University of Zurich, Zurich, Switzerland; 2 HeartClinic Hirslanden, Zurich, Switzerland; 3 University Hospital Zurich, Zurich, Switzerland

A 24-year-old female patient without cardiac symptoms underwent preoperative echocardiography before correction of severe pectus excavatum.

**Fig. 1:**
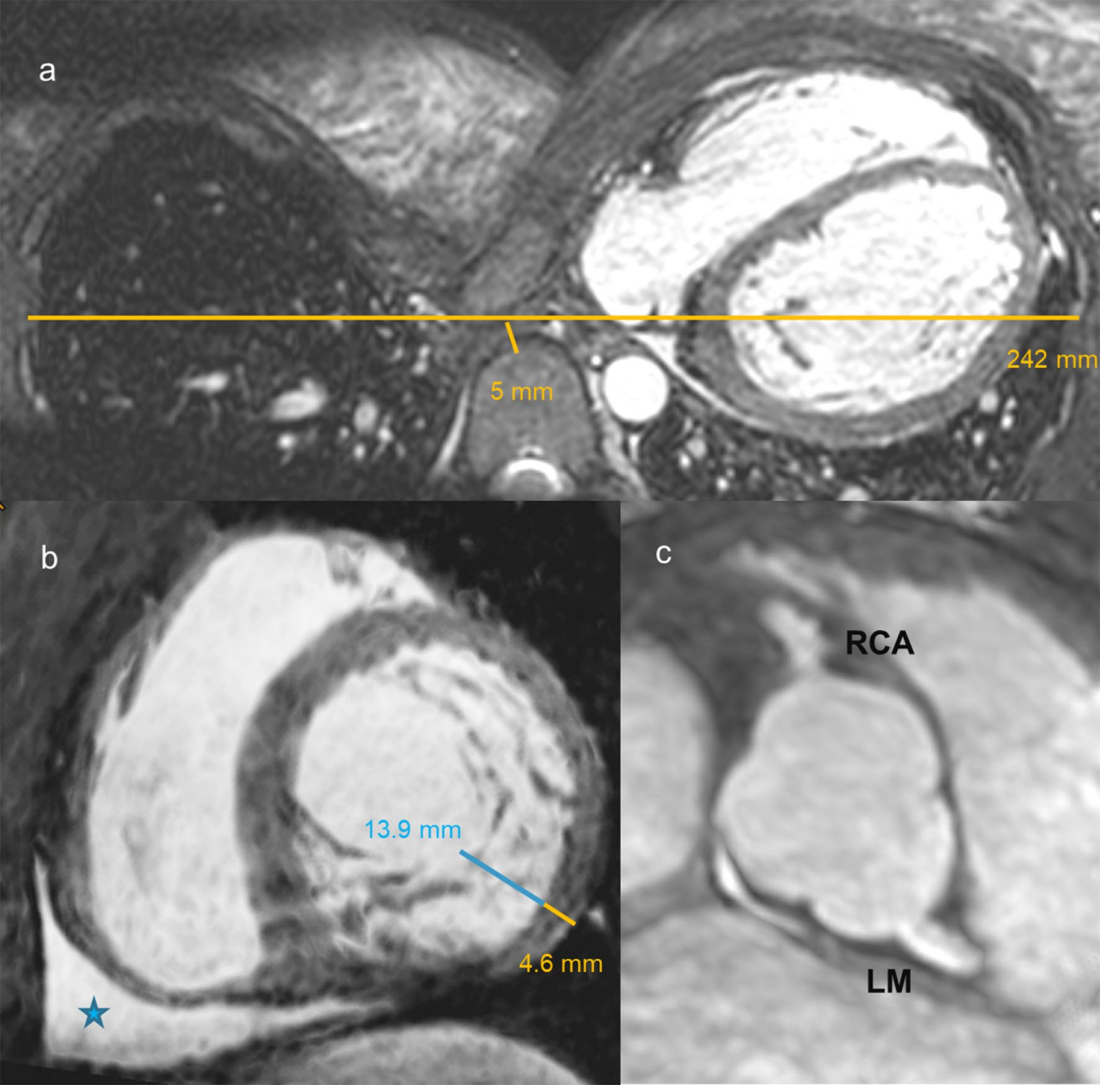
**a** Axial reconstruction of the 3D whole heart sequence demonstrating a severe funnel chest (Haller Index = 48) with mediastinal structures displaced into the left hemithorax. **b** Short-axis stack reconstruction from the 3D whole heart sequence with markedly hypertrabeculated left ventricular wall (ratio of non-compacted/compacted myocardium max. 3.0 in inferolateral midventricular-apical segments). Localized pericardial effusion was detected adjacent to the right ventricular wall (asterisk). **c** In reconstructed maximum intensity projections from the 3D whole heart sequence the sinuses where clearly visible with normal origin of the right (RCA) and left (LM) coronary artery

 Echocardiography was difficult to perform because of the funnel chest, nevertheless, hypertrabeculated apical segments were diagnosed. To confirm the suspicion of non-compaction cardiomyopathy (NCCM) cardiac magnetic resonance imaging (MRI) was performed. For better visualization of non-compacted and compacted myocardium of the left ventricle (LV) a 3-dimensional (3D) whole heart sequence (0.9 mm slice thickness, isotropic resolution) covering the entire heart and adjacent thoracic structures was performed. The 3D whole heart sequence revealed a severe funnel chest (Fig. 1a) with Haller-Index of 48 (transverse diameter of the chest divided by the distance between the anterior surface of the vertebral body and the posterior surface of the sternum, normal ratio < 2). Mediastinal structures were shifted into the left hemithorax. The ratio of non-compacted/compacted (NC/C; normal ratio < 2.3) LV myocardium was up to 3.0 in inferolateral midventricular-apical segments (Fig. 1b). Localized pericardial effusion was detected adjacent to the inferobasal right ventricular wall (Fig. 1b, asterisk). In reconstructed maximum intensity projections from the 3D whole heart sequence the sinuses where clearly visible with normal origin of the right coronary artery (RCA) and the left main coronary artery (LM) (Fig. 1c). With only one whole heart sequence we were able to diagnose several cardiac and non-cardiac pathologies at the same time (funnel chest with quantification of the Haller Index, non-compaction cardiomyopathy with calculation of the NC/C ratio, pericardial effusion) and we visualized a normal origin of the RCA and the LM, since coronary anomalies have been described in NCCM [1].
